# Insulinoma-associated Protein-1 as a Biomarker for Diagnosis of Small Cell Lung Cancer: A Meta-analysis

**DOI:** 10.12669/pjms.42.7.14569

**Published:** 2026-07

**Authors:** Jingtong Zhai, Boyu Qin, Jinzhao Zhai, Jinliang Wang

**Affiliations:** 1 Jingtong Zhai, Senior Department of Oncology, Chinese PLA General Hospital, Beijing 100039, China; 2 Boyu Qin, Senior Department of Oncology, Chinese PLA General Hospital, Beijing 100039, China; 3 Jinzhao Zhai, Senior Department of Oncology, Chinese PLA General Hospital, Beijing 100039, China; 4 Jinliang Wang, Senior Department of Oncology, Chinese PLA General Hospital, Beijing 100039, China

**Keywords:** Insulinoma-associated protein 1, Immunohistochemistry, Pathological diagnosis, Small cell lung cancer

## Abstract

**Objectives:**

Conventional pathological examination can diagnose small cell lung cancer (SCLC) relying on the combined use of multiple immunohistochemical markers, and the diagnostic accuracy still does not meet clinical needs. In recent years, insulinoma-associated protein 1 (INSM1) has gradually demonstrated its value in identifying SCLC in surgical and biopsy specimens of lung tissue. Therefore, we aimed to evaluate the diagnostic utility of INSM1 as an immunohistochemical marker in accelerating and simplifying the diagnosis of SCLC.

**Methodology:**

PubMed, Embase and Web of Science databases were searched to retrieve literature. A meta-analysis was performed with Stata software (Stata 18.0). A random-effects model was used and subgroup analysis was carried out to identify possible sources of heterogeneity.

**Results:**

The initial search identified 257 articles. According to the inclusion criteria, 1523 patients from 10 studies were included in this meta-analysis. The pooled sensitivity and specificity of INSM1 immunohistochemistry for the diagnosis of SCLC were 0.93 (95% CI: 0.89 – 0.96) and 0.96 (95% CI: 0.92 – 0.98), respectively. The corresponding positive likelihood ratio (PLR) was 20.84 (95% CI: 11.64 – 37.32), and the negative likelihood ratio (NLR) was 0.07 (95% CI: 0.04 – 0.12). The area under the summary receiver operating characteristic (SROC) curve was 0.98 (95% CI: 0.97 – 0.99).

**Conclusions:**

INSM1 is a relatively promising and reliable immunohistochemical biomarker for the diagnosis of SCLC, with high sensitivity and specificity. In the future, INSM1 may be widely applied in the diagnosis of lung malignancies to improve the accuracy of pathological diagnosis for SCLC.

## INTRODUCTION

The global burden of lung cancer is extremely heavy.^1^ It is the second most common malignant tumor in humans, accounting for 12% of all new cases of malignant tumors worldwide.^2^ In North American countries, it is the leading cause of cancer-related deaths.^3^ Among all types of lung cancer, non-small cell lung cancer (NSCLC) accounts for the largest proportion and is the primary cause of death.^4^ Small cell lung cancer (SCLC) only constitutes 13-14% of new diagnosed lung malignancies worldwide each year.^5^,^6^ In 2011, there were over 180,000 cases globally, and in 2015, the number exceeded 310,000.^3^,^7^ However, SCLC is the most aggressive type of lung cancer.^8^ Its characteristic is that the tumor cell doubling time is extremely short, and it is more prone to early and extensive metastasis compared to NSCLC.^9^ At present, there is no widely used clinical method for early detection of SCLC, which means that 70% SCLC patients are already in the advanced stage when diagnosed.^10^,^11^ Three-year overall survival rate for patients with SCLC is approximately 56.5% and five-year survival is 10%–20% in most countries.^7^,^12^,^13^ The above data indicate that accurate diagnosis of SCLC is of vital importance.^14^,^15^

At present, the diagnosis of SCLC still relies on immunohistochemical staining of pathological specimens.^16^ However, traditional immunohistochemical markers for lung cancer neuroendocrine tumors, often suffer from low sensitivity and specificity, and even when used in combination, the accuracy are not satisfactory.^17^,^18^ These limitations arise due to issues such as overlap in marker expression between SCLC and other types of lung cancer, as well as variability in diagnostic interpretation. For instance, although IHC is commonly used, its effectiveness can be hindered by weak staining, poor specificity, and the absence of clear-cut positivity thresholds, which can lead to misdiagnoses or delayed treatment. Therefore, in order to achieve an early, rapid and accurate diagnosis of SCLC pathological specimens, a new and more efficient immunohistochemical marker is urgently needed.^13^,^19^

As early diagnosis is crucial for improving outcomes, there is an urgent clinical need for more reliable and specific biomarkers to diagnose lung cancer, particularly SCLC, at an earlier stage. The current diagnostic methods fall short in detecting SCLC accurately in its early stages, which makes the disease harder to treat and worsens patient prognosis. The traditional immunohistochemical biomarkers for lung cancer neuroendocrine tumors include chromogranin A (CGA), synaptophysin (SYP), and CD56. The study by Kosuke et al. demonstrated that INSM1 is a key upstream regulator of the neuronal differentiation pathway in SCLC cell lines, and it was concluded that INSM1 is expressed in 100% of SCLC samples but not expressed in any NSCLC samples.^20^ Besides, a study by Rooper et al. demonstrated that INSM1 was positive in 37 out of 39 cases of SCLC (94.9%), whereas the combined use of conventional markers yielded a positivity rate of only 74.4%. Among non-SCLC specimens, INSM1 expression was observed in 2 of 61 lung adenocarcinomas (3.3%) and 4 of 95 lung squamous cell carcinomas (4.2%), typically exhibiting weak and focal staining patterns. Overall, the sensitivity of INSM1 was 96.4%, which was significantly higher than that of the combined conventional markers (87.4%; P = 0.02). The specificity of INSM1 was 96.2%, comparable to that of traditional markers.^21^ All above indicate that compared to the traditional markers, INSM1 is a more superior neuroendocrine immunohistochemical marker.

In recent years, numerous clinical studies have shown that INSM1 has a high diagnostic ability for SCLC. However, most of the research on INSM1 comes from individual research centers, and the evidence-based medical results from different centers are still lacking. This meta-analysis distinguishes itself by pooling data from multiple independent studies, providing a more comprehensive and robust evaluation of INSM1’s diagnostic value across diverse populations and clinical settings. By conducting a meta-analysis of available studies, we aim to provide robust evidence that supports the clinical application of INSM1 as a diagnostic tool for SCLC. Our objectives are to highlight INSM1’s potential to improve early detection and diagnostic accuracy in clinical practice.

## METHODOLOGY

According to the search strategy, a total of 257 articles were identified from PubMed, Embase, and Web of Science databases. No additional records were found through manual screening of reference lists or grey literature. After removing 105 duplicates, 152 articles were screened by title and abstract. Among these, 114 were excluded due to irrelevance to the topic (n = 104), non-original studies (n = 8), or non-English language (n = 2). Of the 38 articles assessed in full text, four were excluded due to the absence of a control group, 22 lacked sufficient data to construct a 2×2 contingency table, 1 did not use INSM1 for immunohistochemistry, and one was an outdated publication. Finally, 10 studies were included in this meta-analysis.

## RESULTS

### Study characteristics:

A total of 10 studies published between 2017 and 2024 were included in this meta-analysis, with a combined sample size of 1,523 patients (Table-I). These studies were conducted in several countries, including the United States (six studies), Germany one ,Hungary one ,China one ,and Japan one .The types of specimens used for immunohistochemical analysis included surgical specimens in five studies, biopsy samples in four studies, and both surgical and biopsy specimens in 1 study. Regarding antibody clones, 7 studies used INSM1 clone A8 (Santa Cruz), one study used clone A8 (Ventana), 1 used INSM1 MRO-70 (Beijing Zhongshan), and one study did not report the clone type. The cut-off values for positive INSM1 staining were different across studies. Four studies defined positivity as any nuclear staining. Two studies used ≥1% nuclear staining, two used ≥5%, one used ≥10%, and one study used an histological score (*H*-score) cut-off of ≥5. In terms of ethnicity, seven studies focused on Caucasian populations, two studies involved Asian populations, and one study included a mixed population (69% Caucasian, 25% Black, and 3% Asian).

**Table-I:** Main characteristics of 10 studies included in meta-analysis.

Author	Year	Country	Control Type	Ethnicity	Spec-imen Type	Antibody Clone	INSM1 Cut-off	Sample Size	TP	FP	FN	TN
Rooper	2017	America	ADC, SCC	Caucasian	Surgical and Biopsy	INSM1, clone A8 (Santa Cruz)	Any positive nuclear staining	195	37	6	2	150
Doxtader	2018	America	ADC, SCC, mesothelioma	Caucasian	Biopsy	INSM1, clone A8 (Santa Cruz)	Any positive nuclear staining	63	38	0	3	22
Rodriguez	2018	USA	ADC, SCC	Mixed (Caucasian 69%, Black 25%, Asian 3%)	Biopsy	INSM1, clone A8 (Ventana)	≥1% nuclear staining	45	31	0	1	13
Mukhopadhyay	2019	America	ADC, SCC	Caucasian	Surgical	INSM1, clone A8 (Santa Cruz)	≥5% nuclear staining	227	63	5	1	158
Viswanathan	2019	USA	ADC, SCC	Caucasian	Biopsy	INSM1, clone A8 (Santa Cruz)	≥5% nuclear staining	24	8	0	1	15
Kriegsmann	2020	Germany	ADC, SCC	Caucasian	Surgical	INSM1, clone A8 (Santa Cruz)	≥1% nuclear staining	235	124	1	20	90
Sakakibara	2020	Japan	ADC, SCC	Asian	Surgical	INSM1, clone A8 (Santa Cruz)	≥5 H-score nuclear staining	324	72	14	6	232
Tsai	2020	America	Non-NE lung tumors & small round cell tumors	Caucasian	Surgical	INSM1, clone A8 (Santa Cruz)	Any positive nuclear staining	231	40	17	8	166
Zombori	2021	Hungary	ADC, SCC	Caucasian	Surgical	INSM1, clone not specified	Any positive nuclear staining	67	29	7	0	31
Yan	2024	China	ADC, SCC	Asian	Biopsy	INSM1 MRO-70 (Beijing Zhongshan)	≥10% nuclear staining	112	53	3	3	53

ADC: adenocarcinoma; SCC: squamous cell carcinoma; TP: true positive; FP: false positive; FN: false negative; TN: true negative; NE; neuroendocrine.

### Risk of bias and quality assessment:

To assess publication bias, a Deeks’ funnel plot was constructed. The plot exhibited mild asymmetry, with more data points located on the right side of the regression line than on the left. However, the regression line was generally vertical, and the Deeks’ test yielded a p-value greater than 0.10, suggesting that the asymmetry was not statistically significant. While the visual asymmetry could suggest the presence of a small-study effect, where smaller studies with higher diagnostic accuracy might be more likely to be published, the statistical evidence does not support a significant publication bias. Therefore, the risk of publication bias is considered low, though a slight risk cannot be entirely ruled out. According to the methodological quality assessment results of QUADAS-2, the methodological quality of each trial. The quality assessment results of most included studies are acceptable, but in 2 studies, the risk of bias in patient selection is relatively high, which may have an impact on the pooled effect. The overall methodological quality of the included studies rated as moderate to high.

### Diagnostic effect:

**Fig.1:**
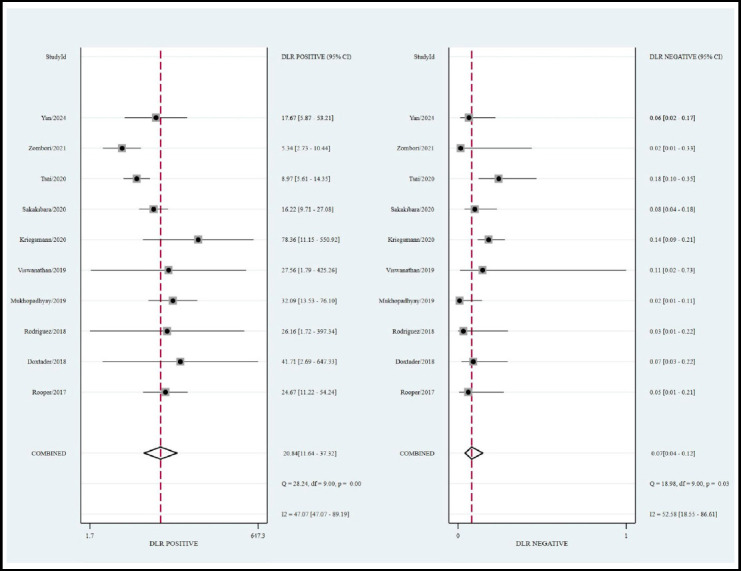
Forest plot of the PLR and NLR for INSM1 in the diagnosis of SCLC. The forest plot shows the pooled positive likelihood ratio (PLR) and negative likelihood ratio (NLR) for INSM1 immunohistochemistry in the diagnosis of SCLC. A higher PLR (20.84) and lower NLR (0.07) indicate the strong ability of INSM1 to both confirm and exclude SCLC. The data were synthesized using a random-effects model, with confidence intervals provided to show the precision of the estimates. The results suggest that INSM1 is a reliable biomarker for diagnosing SCLC with high diagnostic accuracy.

**Fig.2:**
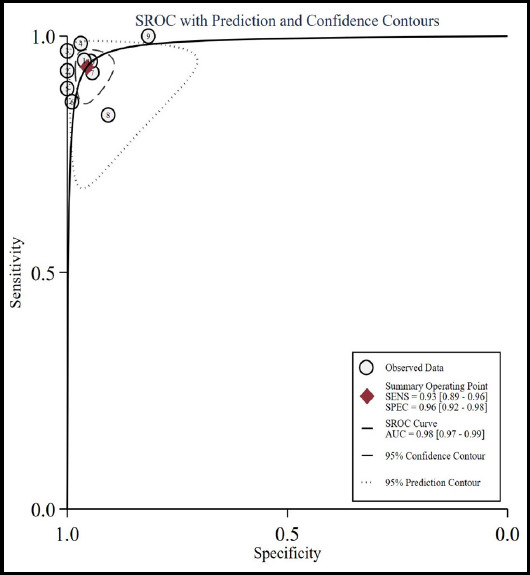
SROC plot of INSM1 for the diagnosis of SCLC. The summary receiver operating characteristic (SROC) curve presents the overall diagnostic performance of INSM1 immunohistochemistry for diagnosing SCLC. The area under the curve (AUC) is 0.98 (95% CI: 0.97–0.99), indicating excellent diagnostic accuracy. A higher AUC represents better diagnostic performance, and in this case, the value close to 1 suggests that INSM1 provides highly accurate results in identifying SCLC across various studies and sample types.

A total of 10 studies involving 1523 patients were included in this meta-analysis. INSM1 immunohistochemistry was evaluated for its diagnostic performance in SCLC. The pooled sensitivity was 0.93 (95% CI: 0.89–0.96), showing a high ability to detect SCLC. The pooled specificity was 0.96 (95% CI: 0.92–0.98), indicating good accuracy in excluding non-SCLC cases. The positive likelihood ratio (PLR) was 20.84 (95% CI: 11.64–37.32), suggesting that a positive INSM1 result was over 20 times more likely in SCLC than in non-SCLC. The negative likelihood ratio (NLR) was 0.07 (95% CI: 0.04–0.12), supporting its strong ability to rule out SCLC when negative (Fig.1). The area under the SROC curve was 0.98 (95% CI: 0.97–0.99), confirming excellent overall diagnostic performance across different studies and sample settings and good stability and reliability (Fig.2).

**Fig.3:**
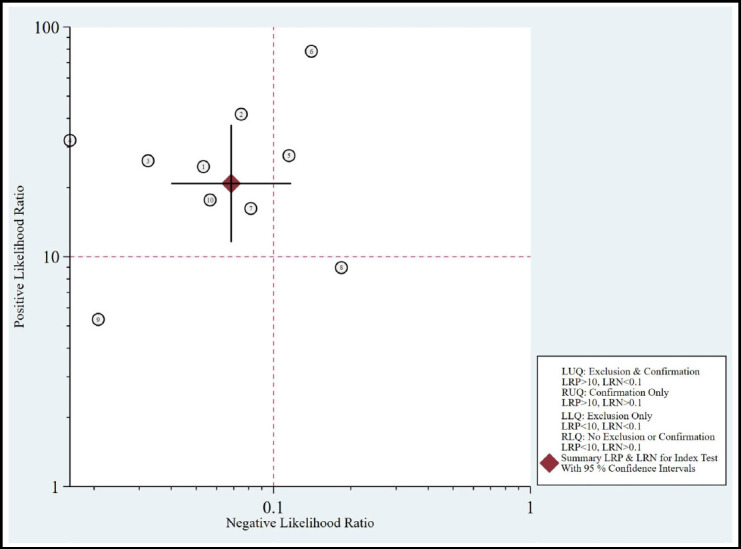
Likelihood ratio scatter-gram evaluating the confirmation and exclusion capacity of INSM1 for diagnosing SCLC. The scatter-gram plots the positive likelihood ratio (PLR) against the negative likelihood ratio (NLR) for INSM1 immunohistochemistry. The point falls in the lower right quadrant, where the PLR is >10 and the NLR is <0.1. This indicates that INSM1 has a strong diagnostic ability for both confirming the presence of SCLC and excluding it when negative. The diagram illustrates the potential of INSM1 to accurately distinguish SCLC from other types of lung cancers and benign conditions.

**Fig.4:**
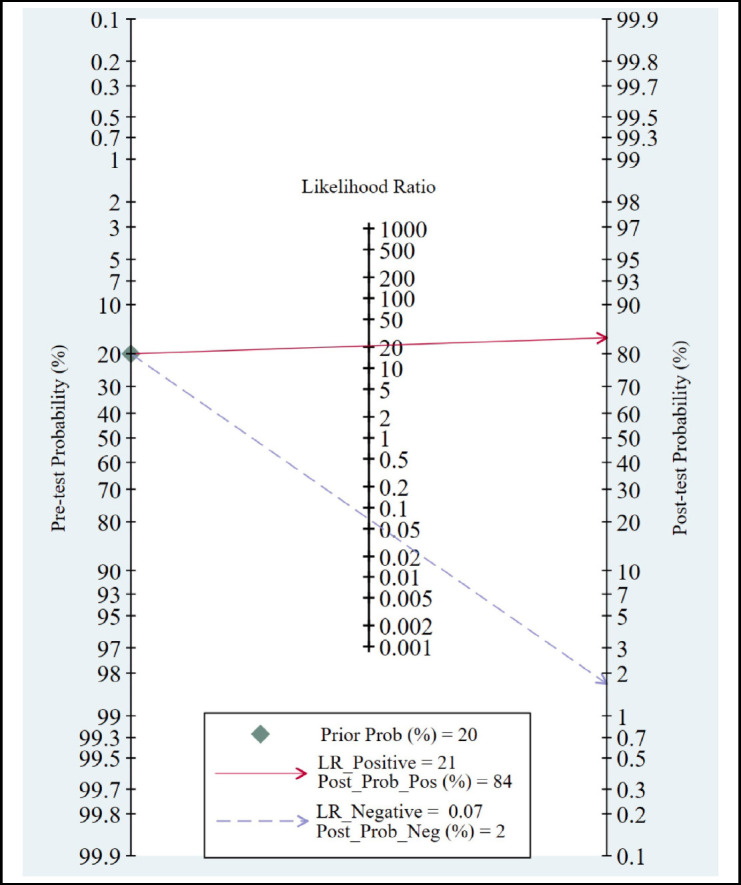
Fagan diagram of INSM1 for diagnosing SCLC. The Fagan diagram demonstrates how INSM1 immunohistochemistry changes the probability of diagnosing SCLC. With a pre-test probability of 20%, a positive INSM1 result increases the post-test probability to 84%, while a negative result reduces it to 2%. This highlights the clinical utility of INSM1 in influencing a physician’s diagnostic judgment, with a significant improvement in diagnostic accuracy based on the test results. The diagram visually conveys the impact of INSM1 on clinical decision-making in the diagnosis of SCLC.

From the likelihood ratio scattergram (Fig.3), the current data points are located in the lower right quadrant (the high PLR, low NLR area), indicating that this test has a good integrative diagnostic ability, and also suggesting that INSM1 immunohistochemical staining has high practical value for the diagnosis of SCLC. Whether used for confirmation or exclusion diagnosis, it shows strong evidential power. Meanwhile, from the Fagan nomogram (Fig.4), the post-test probability corresponding to the current PLR is approximately 85%, and the post-test probability corresponding to the current NLR has decreased to below 2%, indicating that INSM1 can significantly change doctors’ judgment of the patient’s likelihood of illness in clinical practice, and has high clinical translational value for both diagnosis and exclusion. The above results further verify the clinical significance of the high sensitivity and high specificity results in the previous meta-analysis.

## DISCUSSION

This meta-analysis confirms that INSM1 is a highly sensitive and specific immunohistochemical marker for diagnosing SCLC. The pooled sensitivity was 93%, and the specificity was 96%. INSM1 showed excellent diagnostic ability in distinguishing SCLC from non-small cell lung cancer and other similar tumors. The area under the SROC curve (AUC = 0.98) further supports its stable and accurate performance. The clinical impact of INSM1 is significant, as it offers a more reliable alternative to traditional immunohistochemical markers, such as CGA, SYP, and CD56, which have lower sensitivity and specificity. INSM1 demonstrated superior diagnostic performance in both confirming and excluding SCLC, making it a valuable tool in clinical practice for early diagnosis and accurate differentiation of SCLC from other malignancies.

The Santa Cruz A8 clone showed better consistency across studies. INSM1 also maintained stable performance across different populations and specimen types, including biopsy samples. This suggests that INSM1 is widely applicable in clinical settings. Because of its high accuracy, good reproducibility, and ease of use, INSM1 may become a key marker in routine pathological diagnosis of SCLC. Future research should focus on standardizing antibody sources (such as conducting parallel comparison trials of polyclonal antibodies), validating positivity thresholds (such as establishing unified antibody quality control standards), and exploring combined use with other neuroendocrine markers. These efforts will help promote the clinical application of INSM1 in the early and accurate diagnosis of SCLC.

### Author’s contributions:

**ZJ:** Conceived, designed and did statistical analysis and editing of manuscript.

**QB:** Literature search, did data collection and manuscript writing.

**ZJ and WJ:** Did review and final approval of manuscript.

All authors have read, approved the final manuscript and are responsible for the integrity of the study.
